# 基因突变在多发性骨髓瘤预后及治疗中的价值

**DOI:** 10.3760/cma.j.cn121090-20250305-00117

**Published:** 2025-11

**Authors:** 莉莉 程, 俊玲 庄

**Affiliations:** 中国医学科学院、北京协和医学院北京协和医院血液内科，北京 100730 Department of Hematology, Peking Union Medical College Hospital, Chinese Academy of Medical Sciences & Peking Union Medical College, Beijing 100730, China

## Abstract

多发性骨髓瘤（MM）是一种高度异质性血液系统恶性肿瘤，其发病机制涉及复杂的遗传和分子异常。驱动基因突变在MM的发生、发展和预后中起关键作用，主要涉及RAS/MAPK通路、DNA损伤修复通路和NF-κB信号通路。尽管现有危险分层体系已纳入细胞遗传学异常和临床指标，但仍难以精准预测患者预后。近年来，基于基因组异常的预后模型和靶向治疗为MM的精准治疗提供了新方向。未来，结合基因突变分析的预后模型有望进一步改善MM的预后判断和治疗效果。本文对基因突变在MM中的研究现状、预后意义和潜在治疗靶点进行综述。

## Abstract (en)

Multiple myeloma（MM）is a highly het1076erogeneous hematologic malignancy driven by complex genetic and molecular abnormalities. Driver gene mutations, particularly in the RAS/MAPK, DNA damage repair, and NF-κB pathways, are central to MM pathogenesis, progression, and prognosis. Existing risk stratification systems based on cytogenetics and clinical features remain limited in predictive accuracy. Emerging genomic prognostic models and targeted therapies offer new precision treatment strategies. Integrating gene mutation analysis into prognostic frameworks may improve outcome prediction and guide therapy. This review summarizes current advances on gene mutations in MM, their prognostic implications, and potential therapeutic targets.

多发性骨髓瘤（multiple myeloma, MM）是发病率位居第二的血液系统恶性肿瘤，由意义未明单克隆丙种球蛋白病（monoclonal gammopathy of undetermined significance, MGUS）和冒烟型多发性骨髓瘤（smoldering multiple myeloma, SMM）进展而来。MM的生物学和临床特征具有高度异质性[1]，随着新药和治疗策略不断进步，在过去20年间，MM患者的总生存（OS）期已从3～5年延长至8～10年，但MM仍不可治愈，部分高危患者OS期不足3年。因此，精准的危险分层体系对评估MM的预后至关重要。

国际分期系统（ISS）首次提出时仅包含β_2_微球蛋白和血白蛋白两个指标[2]，后续进行过两次修订，增加了高危细胞遗传学异常（HRCA）、乳酸脱氢酶（LDH），并对高危因素进行加权[3]–[4]。尽管HRCA被认为是影响MM患者预后的主要因素，骨髓瘤预后评分系统（MPSS）中还纳入了临床指标如血小板计数等[5]，但MM的发病机制和遗传学异常极其复杂，尤其在新药、新治疗、新检测手段不断涌现的时代，上述危险分层体系难以准确预测患者的生存，需要建立新模型或引入新的预后因素。基因突变在MM发生发展的各个阶段都发挥了重要作用，然而这些突变的预后意义却一直存在争议。本文对基因突变在MM中的研究现状进行综述，并分析其在疾病预后和潜在治疗靶点中的意义。

一、驱动基因突变对疾病演变的影响

肿瘤发生的重要原因之一是基因突变，这些突变因能驱动肿瘤发生而被称为“驱动基因”，它们赋予了体细胞相对于邻近细胞的某些选择优势[6]。2018年发表的一项1 273例新诊断MM（newly diagnosed multiple myeloma, NDMM）的大样本研究中，研究者使用4种方法共发现63个驱动基因，其中84.1％的患者至少包含1种驱动突变，且随着驱动基因突变数量增加，无进展生存（PFS）期和OS期逐渐缩短[7]。驱动基因突变主要发生在RAS/MAPK通路（KRAS、NRAS、BRAF，占56％）、DNA损伤修复通路（TP53、ATM、ATR、BRCA2，占22％）以及NF-κB信号通路（TRAF2、TRAF3、CYLD、NFKB2，占22％）[8]。MM中最常见的驱动基因为RAS突变，分为KRAS（56％）和NRAS（44％）突变[7]，与实体瘤存在差异。这些驱动基因突变常见的发生位点大多也存在差异。其中NRAS突变最常见的位点在Q61密码子上，而KRAS突变最常见位点在G12、G13和Q61密码子上[9]。不同驱动基因间存在一定相关性，如KRAS和NRAS突变互斥，但与BRAF突变却无此关联[8],[10]–[11]。研究MM的驱动基因对于了解肿瘤的生物学特性非常重要，并有助于开发靶向药物。

MM的发生和发展包含多个步骤，由主要和次要遗传事件共同促成[12]。主要事件指染色体异常，发生在B细胞发育早期，是发生MM的启动因素。次要事件通常更为复杂，包括拷贝数异常、MYC易位及驱动基因突变等[1],[13]。虽然染色体异常和驱动基因突变在MM中发生的先后顺序不同，但两者之间存在显著关联，如FGFR3、PRKD2和ACTG1仅在t（4;14）中发生显著突变；CCND1、IRF4、LTB和HUWE1仅在t（11;14）中发生显著突变；MAF仅在t（14;16）中发生显著突变；PRDM1仅在超二倍体中发生显著突变[7]。

驱动基因的突变在MGUS和SMM进展为MM的过程中也发挥了重要作用。2020年的一项SMM基因组特征的研究结果显示，多数NDMM中发现的驱动基因突变在SMM阶段已经出现，并且是疾病进展的独立危险因素。SMM患者如果出现MAPK通路和DNA修复相关基因突变，则进展为MM的风险更高，出现APOBEC相关突变则进展速度更快[14]–[15]。Bustoros等[14]依据这些研究结果提出了第1个基于基因组异常的SMM进展预后评分，包含3个独立风险因素，分别为MAPK信号通路基因突变（KRAS、BRAF、NRAS和PTPN11）、DNA修复异常及MYC易位或拷贝数变异。后来又有研究者在前述研究的基础上对MGUS患者的基因组特征进行分析，发现与SMM结果相似，根据特定的基因组事件可以有效区分两种前体疾病是否稳定[16]。另外，驱动基因突变数量也会影响疾病转化或进展，如突变数量多的SMM患者中位OS期较短，与NDMM的结果相似[7],[17]。KRAS突变在MGUS中很少检测到，但在SMM和MM中很常见，这提示KRAS突变可能与疾病转化有关[17]–[18]。综上，驱动基因突变是MGUS和SMM进展的重要因素，为探究靶向阻断肿瘤进展过程的研究提供了重要基础。

驱动基因突变还与治疗耐药性相关。有研究报道，在难治复发MM（RRMM）中检测到EXZH2、MAML3、TDG、PIGO、NBPF15等10个既往未报道过的潜在驱动基因以及其他新突变，一些基线时已存在的突变在复发时明显增多[19]–[20]。表明新增突变或原有突变可能与耐药或复发有关。一些罕见的驱动基因，如CRBN、IKZF1或IKZF3突变可预测对免疫调节剂的耐药性[21]，XBP1突变可预测对硼替佐米的耐药性[7]。

二、基因突变在MM中的预后意义

MM属于惰性肿瘤，从克隆性浆细胞发生、肿瘤负荷增多至产生终末器官损伤常需要数年甚至十余年[22]。因此，很多在癌前病变时发生的基因突变会一直延续到需要治疗的MM阶段，并在疾病进展、病灶迁移、耐药等方面继续发挥作用。

虽然MM中能检出的基因突变很多，但多数预后意义并不确定，只有少数突变与预后不良存在显著相关性。其中，最为明确的是TP53突变[23]。在NDMM中，TP53异常分为以下三类：发生17p−的单等位基因缺失（占8％）、单等位基因突变（占6％）和双等位基因失活（占4％）。其中17p−是多种MM分期标准中已确定的高风险特征之一。TP53突变在MM早期进展中发挥了重要作用[24]。国内最新的高危MM（HRMM）专家共识将初治和复发时新出现TP53突变纳入HRMM诊断标准[25]。但因其作用机制复杂，暂无靶向TP53的药物应用于临床[26]。

RAS突变是NDMM中突变频率最高的基因，在RRMM中的突变频率更高[27]，突变发生在非典型密码子如A146、K117和Q22更为常见[28]。在实体瘤中，RAS突变与不良预后明确相关，但在MM中尚无定论。多数研究发现，其与更短的PFS期和OS期相关[28]，并促使髓外病变发生，导致疾病更为难治[29]。但也有研究认为，RAS突变并不影响MM的整体预后，其中最主要的两种类型是KRAS和NRAS[9],[30]。BRAF也是RAS突变的一种，比例稍低于KRAS和NRAS[7]。此外，最常见的突变还包括FAM46C和DIS3，在MM中发生比例仅次于RAS突变[7]。多个研究显示，FAM46C和DIS3也与肿瘤发生发展密切相关（表1）。

**表1:** 多发性骨髓瘤中常见的基因突变

突变基因	信号通路	突变频率[7]	与肿瘤发生发展的相关性
KRAS	RAS/MAPK	22％	结论不一，多数呈负相关[9],[29]–[32]
NRAS	RAS/MAPK	18％	结论不一，多数呈负相关[9],[29]–[32]
DIS3	RNA代谢[33]	10％	呈负相关[34]–[35]
FAM46C	PI3K-Akt[36]	10％	呈负相关[36]–[38]
BRAF	RAS/MAPK	8％	结论不一，多数呈负相关[9],[29]–[32]
TP53	DNA修复	6％	呈负相关[23]

除上述较为常见的驱动基因突变外，IGLL5被发现与血液系统恶性肿瘤发生增加有关[39]。该基因与MM疾病进展有关，在NDMM中的突变频率为16％～18％，且与各种RAS突变存在互斥[24],[40]。IGLL5是MM早期进展的独立危险因素，与一年后进展患者相比，一年内进展患者的突变发生率较高（20％对14％）[24]。

应用较为广泛的MM预后系统以ISS及两次修订的标准为代表[2]–[4]，还包括结合基因组学建立的IRMMa模型及预测早期复发的S-ERMM模型等[41]–[42]，但鲜有将基因突变纳入预后分层的标准。近年的研究发现，部分MM患者基线时没有任何已知的高风险遗传因素，但在早期（12～18个月）就发生疾病进展，且预后极差，称为功能性高危（functional high-risk, FHR）MM[42]。二代测序结果显示，KIAA1549L、LUZP2和BMPR1B等基因突变会特异发生在FHR MM患者中，影响IL-6/JAK/STAT3通路的突变也明显增多，说明现有危险分层系统未识别出高危患者可能是未知基因突变所致[43]。目前的危险度分层体系不能精准预测MM患者的预后，基于MM发病机制的复杂性，可能需要纳入更全面的预后因素如基因突变，建立适应更复杂情况的预测模型才能不断提高预后判断的准确性。

三、基因突变与靶向治疗

尽管IRMMa预测模型的预后因素包含基因突变[43]，但目前根据基因突变指导MM治疗的研究仍十分有限。RAS/MAPK通路上重要的基因突变可以通过影响细胞增殖、存活和分化在MM中发挥关键作用，因此成为靶向治疗研究的主要信号通路之一[43]–[44]。AZD4785是一种高效、选择性靶向KRAS的反义寡核苷酸，能与KRAS的3′端非翻译区结合，靶向所有KRAS突变亚型，特异性沉默KRAS并在体内和体外显著抑制肿瘤细胞生长[27]，具有良好的治疗潜力。CH5126766/VS-6766是一种新型MEK-pan-RAF抑制剂，通过间歇性给药方式，在携带RAS-RAF-MEK通路突变的MM患者中表现出良好且持久的缓解及良好的耐受性[45]。另一项基础研究发现，GCK抑制剂TL4-12可诱导RAS突变的MM细胞出现细胞周期停滞并促进细胞凋亡，联合来那度胺治疗时可以发挥协同作用[46]（表2）。

**表2:** 靶向基因突变治疗多发性骨髓瘤（MM）的药物总结

药物名称	靶向突变	药物类型	作用效果
AZD4785[27]	KRAS	反义寡核苷酸	体内和体外抑制MM肿瘤细胞生长
CH5126766/VS-6766[45]	KRAS	MEK-pan-RAF抑制剂	客观反应率为27％
TL4-12[46]	RAS（NRAS、KRAS、BRAF）	GCK抑制剂	促进细胞周期停滞和细胞凋亡，降低对免疫调节剂的耐药性
恩考芬尼联合比美替尼[47]	BRAFV600E	BRAF/MEK抑制剂	中位PFS期5.6个月，24个月OS率55％
达拉非尼联合曲美替尼[48]	BRAFV600E	BRAF/MEK抑制剂	中位DOR 11.1个月，中位PFS期6.3个月，中位OS期33.9个月

**注** PFS：无进展生存；OS：总生存；DOR：缓解持续时间

RAS/MAPK通路的另一个常用的靶向药物为BRAF抑制剂。一项多中心Ⅱ期试验评估BRAF/MEK抑制剂恩考芬尼和比美替尼联合应用于BRAFV600E突变RRMM患者的疗效。结果显示，总体有效率为83.3％，中位PFS期为5.6个月，24个月OS率为55％，这是首个证明联合BRAF/MEK抑制剂对BRAFV600E突变RRMM患者有效的前瞻性临床试验[47]。Ⅱ期ROAR试验评估了达拉非尼（BRAF激酶抑制剂）联合曲美替尼（MEK抑制剂）对BRAFV600E突变RRMM患者的疗效和安全性，结果显示，总体有效率为50％，中位缓解持续时间为11.1个月，中位PFS期为6.3个月，中位OS期为33.9个月[48]（表2）。靶向分子及信号通路的治疗可以在有效杀死肿瘤细胞的同时保护健康细胞，最大限度地减少不良反应，可作为有特定基因突变患者的治疗选择。但靶向治疗也存在一定的局限性，如多数中心并未开展二代测序，且有些突变的发生率低。另一个局限在于，靶向基因突变的治疗并未考虑RNA和蛋白质水平的变化，而很多药物主要作用于蛋白质，因此治疗结果可能并不稳定[49]。

MM的发病机制和疾病异质性显著，目前的预后分层体系仍不能精准预测临床结局。随着对MM的认识逐渐深入，治疗策略不断突破，危险分层体系也在不断完善。尽管临床指标和细胞遗传学异常是主要危险因素，未来对基因突变的分析仍有很大空间，突变异常可能成为预后模型的有力补充，从而更好地预测疾病转归。
